# Alcohol Consumption and Age-Specific Risk of Esophageal Cancer: Prospective Cohort Study

**DOI:** 10.2196/92949

**Published:** 2026-06-03

**Authors:** Yongbo Yang, Xing Xing, Zhen Qin, Chunyang Han, He Zhu

**Affiliations:** 1 First Department of Thoracic Surgery, Peking University Cancer Hospital and Institute, Beijing, China; 2 Key Laboratory of Carcinogenesis and Translational Research, Ministry of Education, Peking University Cancer Hospital and Institute, Beijing, China; 3 School of Public Health, Peking University, 38 Xueyuan Rd, Haidian District, Beijing, 100191, China, 86 010 82805755; 4 The First Clinical School, Huazhong University of Science and Technology, Wuhan, Hubei, China; 5 National Health Commission Key Laboratory of Health System Reform and Governance (Peking University), Peking University, Beijing, China; 6 Beijing Institute for Health Development, Peking University, Beijing, China

**Keywords:** esophageal cancer, age, alcohol, China Kadoorie Biobank, cohort study, age-specific association

## Abstract

**Background:**

China accounts for more than 40% of new global cases and deaths from esophageal cancer, and has a relatively high rate of past-year alcohol use, reaching up to 27%. The incidence and risk factors of esophageal cancer exhibit marked age-related variation; however, the impact of alcohol consumption on the risk of esophageal cancer across different age groups remains poorly understood.

**Objective:**

This study aimed to investigate the age-specific associations between alcohol consumption and the risk of esophageal cancer.

**Methods:**

Data were obtained from the China Kadoorie Biobank, a large-scale, nationwide prospective cohort study. The final analysis included 489,664 adult participants aged 30 to 79 years enrolled at baseline between 2004 and 2008. Age-specific incidence rates per 100,000 person-years of esophageal cancer were calculated. Cox proportional hazards models were used to assess the associations between alcohol consumption and the risk of esophageal cancer, stratified by age group.

**Results:**

In the study cohort, incidence rates of esophageal cancer consistently increased with higher frequency of alcohol consumption among all age groups younger than 70 years. The association exhibited an age-specific pattern: within each age group, the rate ratio for weekly versus never drinkers was highest in the youngest group (8.31 for the 30‐49 year age group) and declined with age; across age groups, among weekly drinkers, the incidence rate increased sharply from the 30‐49 year to the 50‐54 year age group (rate ratio 2.81). Within each age group, compared with never drinkers, the adjusted hazard ratios for weekly drinkers decreased progressively with age group, from 4.06 (95% CI 2.73‐6.02) in adults aged 30-49 years to 3.17 (95% CI 2.34-4.30), 2.88 (95% CI 2.20-3.77), 2.36 (95% CI 1.78-3.14), 2.25 (95% CI 1.69-3.00), and 1.61 (95% CI 1.15-2.25) in those aged 50‐54, 55‐59, 60‐64, 65‐69, and 70‐74 years, respectively.

**Conclusions:**

Our findings highlight a potential age-modifying effect of alcohol consumption on the risk of esophageal cancer, with a strong relative risk observed in young adults and a marked acceleration during the transition to midlife. This underscores the need to develop age-specific public health strategies to reduce alcohol use and to strengthen screening and intervention efforts to reduce the burden of esophageal cancer.

## Introduction

Esophageal cancer is a highly aggressive malignancy characterized by a poor prognosis, reduced quality of life, and low survival rates, representing a substantial global health burden [1]. According to the Global Cancer Observatory estimates, esophageal cancer was the 11th most commonly diagnosed cancer, with 2.6% of all new cancer cases and the seventh leading cause of cancer-related deaths, accounting for 4.6% of all cancer deaths worldwide in 2022 [2]. Notably, in 2022, China accounted for 43.8% of new global cases and 42.1% of deaths from esophageal cancer [3], and an age-standardized incidence rate of 8.32 per 100,000 population and an age-standardized mortality rate of 6.68 per 100,000 population were higher than the global average, ranking as the seventh most prevalent cancer and the fifth leading cause of cancer-related death within the country [4]. Furthermore, most patients are diagnosed at advanced stages, and esophageal cancer is associated with dysphagia and weight loss, reduced functional status, increased comorbidities, higher risk of treatment-related toxicities from aggressive multimodal therapies, and increased mortality risk [1,5,6]. Given this substantial clinical and economic burden, there is a clear need to better understand the underlying risk factors to inform early detection and targeted intervention efforts.

Esophageal cancer exhibits pronounced age-related characteristics, and the risk increases significantly with age, with a particularly high prevalence among older adults [1,7]. However, recent estimates indicate a concerning increase in incidence rates among younger populations [8]. National and global data indicate that incidence rates generally peak between the ages of 70 and 85 years [5,9-11]. An analysis of Chinese national data from 2005 to 2015 found that age is an independent risk factor for esophageal cancer; the risk of developing the disease increases significantly, with an odds ratio of 1.18 for each additional year of age and an odds ratio of 1.62 for every additional 5 years [12]. Evidence suggests that the impact of certain risk factors may vary across different age groups; however, these variations across the age spectrum remain poorly understood. For example, a study using large-scale pooled data from 8 population-based case-control studies within the international Barrett’s and Esophageal Adenocarcinoma Consortium (BEACON) revealed that smoking and reflux were positively associated with an increased risk of esophageal adenocarcinoma (EAC) in all age groups, but obesity and recurrent gastroesophageal reflux were significantly stronger risk factors for individuals diagnosed before the age of 50 compared with older age groups [8]. There is a clear need to investigate age-specific risk factors for esophageal cancer to better inform targeted prevention strategies for differential susceptibility.

Alcohol consumption is a major modifiable risk factor for esophageal cancer, particularly for esophageal squamous cell carcinoma (ESCC), which is the predominant histological subtype accounting for approximately 90% of cases in China [13]. This differs from the Western population, where EAC is more common and exhibits a distinct etiological profile [2,14]. Moreover, alcohol consumption warrants particular attention due to its high prevalence in the Chinese population, and the recent national data reported that 27.6% of individuals aged 15 years and older consumed alcohol in the past year in China, with this rate reaching a striking 44.5% among men [15]. However, the impact of alcohol on esophageal cancer risk across the lifespan remains poorly characterized, particularly in Chinese populations. One study, largely derived from Western studies, suggested that alcohol shows little to no association with EAC risk among individuals aged <50 years and those in the 50‐59 and 60‐69 year age groups [8]. In contrast, research on ESCC demonstrated a monotonic increase in risk associated with younger age at drinking initiation, prolonged duration, and higher intensity of alcohol intake [16]. Furthermore, the age-specific etiological role of alcohol across the lifespan remains poorly characterized, largely due to historical sample size limitations. Because early-onset esophageal malignancies are relatively rare, previous studies assessing risk factors have often been constrained by small sample sizes, precluding detailed and statistically robust investigations of alcohol consumption across multiple distinct age groups [8]. Therefore, there is a clear need to explore large-scale, population-based data to systematically examine the age-specific associations between alcohol consumption and esophageal cancer risk to add evidence for the Chinese population.

Therefore, this study sought to comprehensively investigate the associations between alcohol consumption and the risk of esophageal cancer across various age groups using a cohort of 0.5 million individuals in China. The findings are expected to provide robust evidence to inform the development of more precise, efficient, and cost-effective risk-stratified screening strategies to prevent and intervene against esophageal cancer in China.

## Methods

### Data Source and Study Cohort

The data were derived from the China Kadoorie Biobank (CKB) resource (request numbers DAR-2025‐00208 and DAR-2025‐00316). The CKB is a large-scale, nationwide prospective cohort study designed to investigate the genetic and environmental determinants of major chronic diseases in China, established across 10 geographically diverse research sites, including 5 urban and 5 rural areas [17-19]. The selection of 10 sites considered the prevalence of chronic diseases, exposure profiles, economic development, stability of the local population, and local capacity to capture diverse disease patterns and risk exposures. At baseline, more than 512,000 adults aged 30 to 79 years were enrolled between 2004 and 2008, and demographic characteristics, socioeconomic status, lifestyle habits (including smoking, alcohol consumption, diet, and physical activity), and medical history were collected via an interviewer-administered electronic questionnaire. At the follow-up, the vital status and incident disease events of the cohort participants were continuously monitored through established linkages with regional death registries, disease surveillance systems, and national health insurance claim databases. Publication of results does not require or imply approval by the membership of the CKB Collaborative Group.

In this study, the data, including a total of 512,724 participants, were obtained from the CKB, and we excluded participants with a prior diagnosis of any cancer at baseline (n=2578, 0.5%), those with missing family history information (n=20,480, 3.99%), and those with missing BMI data (n=2, 0%). After these exclusions, a total of 489,664 (95.5%) participants were included in the final analysis. To examine age-related differences, all participants were categorized based on their baseline age into the following groups: 30‐49 years (n=223,074, 45.55%), 50‐54 years (n=84,708, 17.3%), 55‐59 years (n=66,865, 13.66%), 60‐64 years (n=46,663, 9.53%), 65‐69 years (n=38,344, 7.83%), 70‐74 years (n=26,862, 5.49%), and 75‐79 years (n=3148, 0.64%). The 5-year interval was selected because it allows for a more granular exploration of age-specific associations between alcohol consumption and esophageal cancer risk, captures potential nonlinear patterns, and improves direct comparisons with previous studies that widely used similar age-specific groupings [10,20]. Additionally, we grouped ages 30‐49 years into one age group mainly due to the relatively low incidence of esophageal cancer among individuals younger than 50 years in the CKB data, thereby avoiding sparse data and limited statistical power and ensuring sufficient case numbers and statistical robustness for the regression models examining alcohol-related risks.

### Study Variables

#### Esophageal Cancer

The primary outcome of this analysis was esophageal cancer, defined according to the *International Classification of Diseases, Tenth Revision* (*ICD-10*) code C15. Cases of esophageal cancer were identified through linkage with multiple sources by the CKB, including regional cancer registries, mortality databases, and the national health insurance system. To ensure comprehensive case identification and minimize loss to follow-up, all disease outcomes were coded by trained research staff, and annual active surveillance was performed using residential records, health insurance data, and administrative databases, with additional verification through direct contact with participants or their relatives when necessary [18].

#### Alcohol Consumption at Baseline

Information on alcohol consumption habits was collected from CKB participants using a standardized questionnaire during the baseline survey. Participants were asked about their drinking frequency over the past 12 months, which was categorized as follows: (1) never drinking (ie, never or almost never), (2) only occasionally (ie, only occasionally or only at certain seasons), (3) every month but less than weekly, and (4) at least once a week or weekly drinking [21]. This variable was used as the exposure in this study. Furthermore, participants who reported drinking at least once a week were additionally asked about their age at initiation of alcohol consumption. This was further categorized into 5 groups: <15, 15‐24, 25‐34, 35‐44, and ≥45 years to examine the associations between the age of alcohol initiation and the risk of esophageal cancer among weekly drinkers.

#### Covariates at Baseline

To reduce the possible confounding influences related to the estimated association, covariates were selected mainly in accordance with the risk factors identified in previous studies [13,16,22-24] and the availability of the CKB database at baseline, including (1) demographic variables: age group, sex (male or female), education level (uneducated, primary school, middle school, high school, technical school or college, or university), household income (≤¥4999, ¥5000‐¥19,999, or ≥¥20,000; an exchange rate of ¥1=US $0.15 is applicable), area (rural or urban), and marital status (unmarried, married, or divorced or widowed); (2) lifestyle factors: physical activity (low, medium, or high), BMI (normal, underweight, overweight, or obese), smoking status (did not smoke, only occasionally, on most days, or daily), and family history of cancer (no or yes). In detail, BMI was calculated using the standard formula: weight (kg)/height (m)^2^, derived from objective measurements of weight and height. Body weight and height were measured by trained staff using calibrated instruments. Physical activity was defined using metabolic equivalent task hours per day (MET-h/d) spent on work, transportation, housework, and nonsedentary recreation, and participants were then categorized into low, medium, or high groups based on the 25th and 75th percentiles.

### Statistical Analysis

First, we described demographic characteristics and lifestyle factors stratified by alcohol consumption subgroups. Categorical variables were presented as percentages and compared using chi-square tests. Second, we calculated age-specific incidence rates (per 100,000 person-years) of esophageal cancer by baseline age group and alcohol consumption subgroup. For each age stratum, person-years were accrued from the baseline date until the date of esophageal cancer diagnosis, loss to follow-up, or December 31, 2018, whichever occurred first, stratified by alcohol consumption subgroup. Rates were then computed within each stratum and alcohol consumption category. Third, Cox proportional hazards models were used to evaluate the association between alcohol consumption and the risk of esophageal cancer in the total sample and across different age groups, after adjusting for sex, education level, household income, area, marital status, smoking status, physical activity, BMI, and family history of cancer, and across sex and age groups after adjusting for these covariates. For further analysis among weekly drinkers, we used Cox models to examine the association between age at alcohol initiation and esophageal cancer risk, adjusting for the same set of covariates. The proportional hazards assumption was tested using Schoenfeld residuals. Results are presented as hazard ratios with corresponding 95% CIs. Finally, to address potential selection bias arising from our exclusion criteria, we conducted a sensitivity analysis using inverse probability weighting. We modeled the probability of inclusion using logistic regression based on demographic characteristics and applied these probabilities as weights in our Cox proportional hazards models. All statistical analyses were performed using R (version 4.4.2; R Foundation for Statistical Computing).

### Ethical Considerations

Ethical approvals for the CKB were obtained from Oxford Tropical Research Ethics Committee (025‐04) at the University of Oxford, Oxford, United Kingdom, and the Ethical Review Committee from the Chinese Center for Disease Control and Prevention (005/2004), Beijing, China. All participants provided written informed consent upon recruitment into the CKB. This study, which used CKB data to examine the association between alcohol consumption and the risk of esophageal cancer, was approved by the institutional review board of Peking University, Beijing, China (IRB00001052-25128).

## Results

### Baseline Characteristics of the Study Cohort

**Figure 1.**
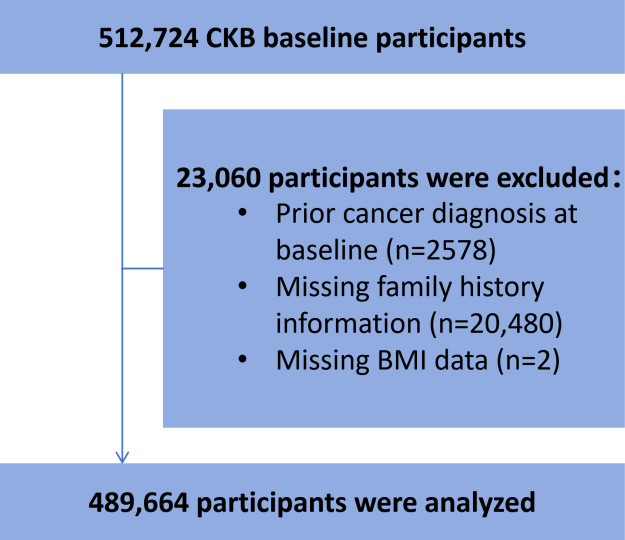
Flow diagram of the study cohort selection. CKB: China Kadoorie Biobank.

Overall, of the 489,664 participants (Figure 1), the study cohort was predominantly female (n=291,555, 59.54%), aged 30 to 49 years (n=223,074, 45.56%), had primary school education (n=156,853, 32.03%), were married (n=448,075, 91.51%), resided in rural areas (n=273,376, 55.83%), and reported a medium income level (n=232,737, 47.53%) at baseline (Table 1).

Most participants had a normal BMI (n=255,030, 52.08%), did not smoke (n=347,209, 70.91%), engaged in high levels of physical activity (n=167,952, 34.30%), and had no family history of cancer (n=403,815, 82.47%).

Regarding alcohol consumption at baseline, of the total sample (n=489,664), 233,038 (47.59%) reported never drinking, 165,137 (33.72%) drank only occasionally, 18,908 (3.86%) drank every month but less than weekly, and 72,581 (14.82%) drank at least once a week. Participants aged 30 to 49 years accounted for more than half of the monthly and weekly drinkers. All subgroups differed significantly across alcohol consumption categories (*P*<.001).

**Table 1.** Characteristics of the study cohort stratified by alcohol consumption.

Variables	Overall (n=489,664), n (%)	Never (n=233,038), n (%)	Only occasionally (n=165,137), n (%)	Every month but less than weekly (n=18,908), n (%)	At least once a week (n=72,581), n (%)	*P* value
Age group (years)						<.001
30‐49	223,074 (45.56)	95,708 (41.07)	82,713 (50.09)	10,917 (57.74)	33,736 (46.48)	
50‐54	84,708 (17.30)	39,648 (17.01)	28,474 (17.24)	3108 (16.44)	13,478 (18.57)	
55‐59	66,865 (13.66)	34,026 (14.60)	20,985 (12.71)	2065 (10.92)	9789 (13.49)	
60‐64	46,663 (9.53)	25,041 (10.75)	13,866 (8.40)	1223 (6.47)	6533 (9.00)	
65‐69	38,344 (7.83)	21,455 (9.21)	10,832 (6.56)	885 (4.68)	5172 (7.13)	
70‐74	26,862 (5.49)	15,343 (6.58)	7366 (4.46)	642 (3.40)	3511 (4.84)	
75‐79	3148 (0.64)	1817 (0.78)	901 (0.55)	68 (0.36)	362 (0.50)	
Sex						<.001
Male	198,109 (40.46)	46,647 (20.02)	70,203 (42.51)	14,654 (77.50)	66,605 (91.77)	
Female	291,555 (59.54)	186,391 (79.98)	94,934 (57.49)	4254 (22.50)	5976 (8.23)	
Education						<.001
Uneducated	89,927 (18.37)	65,710 (28.20)	17,038 (10.32)	1053 (5.57)	6126 (8.44)	
Primary school	156,853 (32.03)	81,850 (35.12)	47,640 (28.85)	4374 (23.13)	22,989 (31.67)	
Middle school	140,126 (28.62)	55,404 (23.77)	53,630 (32.48)	6618 (35.00)	24,474 (33.72)	
High school	74,528 (15.22)	23,709 (10.17)	33,064 (20.02)	4580 (24.22)	13,175 (18.15)	
Technical school or college	17,248 (3.52)	3955 (1.70)	8506 (5.15)	1306 (6.91)	3481 (4.80)	
University	10,982 (2.24)	2410 (1.03)	5259 (3.18)	977 (5.17)	2336 (3.22)	
Income (¥)^a^						<.001
≤4999	45,913 (9.38)	24,438 (10.49)	15,064 (9.12)	1057 (5.59)	5354 (7.38)	
5000‐19,999	232,737 (47.53)	108,756 (46.67)	85,129 (51.55)	8722 (46.13)	30,130 (41.51)	
≥20,000	211,014 (43.09)	99,844 (42.84)	64,944 (39.33)	9129 (48.28)	37,097 (51.11)	
Area						<.001
Rural	273,376 (55.83)	137,325 (58.93)	89,727 (54.33)	10,020 (52.99)	36,304 (50.02)	
Urban	216,288 (44.17)	95,713 (41.07)	75,410 (45.67)	8888 (47.01)	36,277 (49.98)	
Marital status						<.001
Unmarried	554 (0.11)	211 (0.09)	199 (0.12)	37 (0.20)	107 (0.15)	
Married	448,075 (91.51)	209,335 (89.83)	152,550 (92.38)	17,770 (93.98)	68,420 (94.27)	
Divorced or widowed	41,035 (8.38)	23,492 (10.08)	12,388 (7.50)	1101 (5.82)	4054 (5.59)	
BMI						<.001
Normal	255,030 (52.08)	123,177 (52.86)	84,375 (51.09)	9572 (50.62)	37,906 (52.23)	
Underweight	21,528 (4.40)	12,850 (5.51)	5656 (3.43)	528 (2.79)	2494 (3.44)	
Overweight	162,015 (33.09)	73,401 (31.50)	56,865 (34.44)	6801 (35.97)	24,948 (34.37)	
Obese	51,091 (10.43)	23,610 (10.13)	18,241 (11.05)	2007 (10.61)	7233 (9.97)	
Smoking						<.001
Did not smoke	347,209 (70.91)	202,886 (87.06)	115,472 (69.92)	7992 (42.27)	20,859 (28.74)	
Only occasionally	21,637 (4.42)	3612 (1.55)	10,474 (6.34)	2035 (10.76)	5516 (7.60)	
On most days	3580 (0.73)	712 (0.31)	1358 (0.82)	410 (2.17)	1100 (1.52)	
Daily	117,238 (23.94)	25,828 (11.08)	37,833 (22.91)	8471 (44.80)	45,106 (62.15)	
Physical activity						<.001
Low	160,732 (32.82)	81,026 (34.77)	54,537 (33.03)	5252 (27.78)	19,917 (27.44)	
Medium	160,980 (32.88)	73,300 (31.45)	57,726 (34.96)	6203 (32.81)	23,751 (32.72)	
High	167,952 (34.30)	78,712 (33.78)	52,874 (32.02)	7453 (39.42)	28,913 (39.84)	
Family history of cancer						<.001
No	403,815 (82.47)	197,866 (84.91)	132,350 (80.15)	15,052 (79.61)	58,547 (80.66)	
Yes	85,849 (17.53)	35,172 (15.09)	32,787 (19.85)	3856 (20.39)	14,034 (19.34)	

a An exchange rate of ¥1=US $0.15 is applicable.

### Incidence Rates of Esophageal Cancer Stratified by Age Group and Alcohol Consumption at Baseline

**Figure 2.**
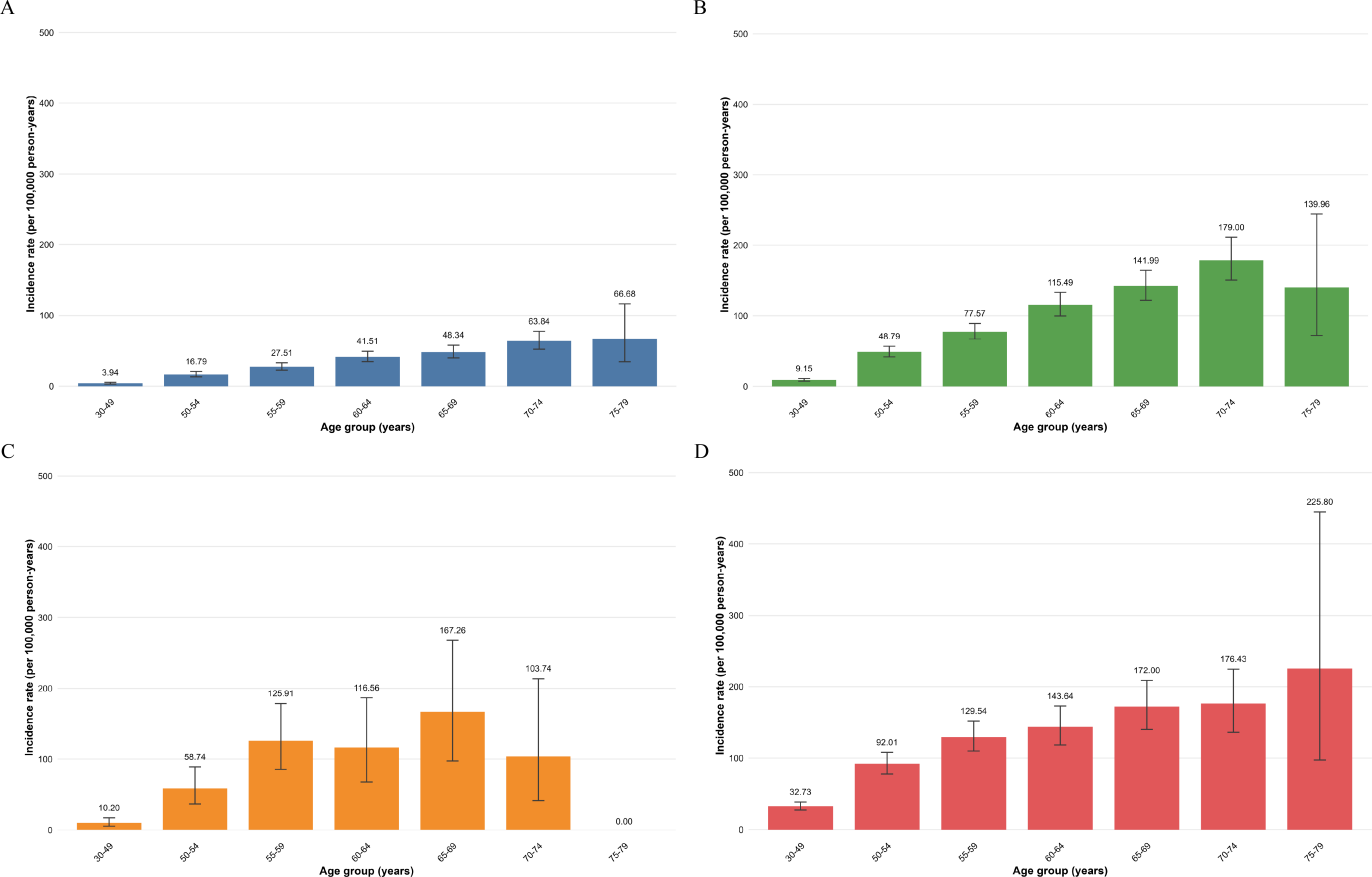
Incidence rates per 100,000 person-years of esophageal cancer stratified by age group and alcohol consumption: (A) never, (B) only occasionally, (C) every month but less than weekly, and (D) at least once a week.

Within each age stratum, incidence rates consistently increased with higher frequency of alcohol consumption among age groups younger than 70 years (Figure 2). The incidence rate ratio comparing weekly drinkers to never drinkers was highest in the youngest age group and progressively declined with advancing age. Specifically, the rate ratio was 8.31 in the 30‐49 year age group, decreasing to 5.48 in the 50‐54 year age group, 4.71 in the 55‐59 year age group, 3.46 in the 60‐64 year age group, 3.56 in the 65‐69 year age group, 2.76 in the 70‐74 year age group, and 3.39 in the 75‐79 year age group.

Across age groups, among weekly drinkers, the rate ratio comparing successive age groups was 2.81 for ages 50‐54 vs 30‐49 years (92.01/32.73), 1.41 for ages 55‐59 vs 50‐54 years (129.54/92.01), 1.11 for ages 60‐64 vs 55‐59 years (143.64/129.54), 1.20 for ages 65‐69 vs 60‐64 years (172.00/143.64), 1.03 for ages 70‐74 vs 65‐69 years (176.43/172.00), and 1.28 for ages 75‐79 vs 70‐74 years (225.80/176.43). Because of the small number of participants in the 75‐79 year age group, these findings should be considered as preliminary results.

### Alcohol Consumption and the Risk of Esophageal Cancer in the Overall Study Cohort

In the overall study cohort, after adjusting for covariates, all levels of alcohol consumption were significantly associated with an increased risk of esophageal cancer (all *P*<.001; Table 2). Compared with never drinkers, the adjusted hazard ratios (aHRs) were 2.02 (95% CI 1.82‐2.24) for participants who drank only occasionally, 1.75 (95% CI 1.41‐2.18) for those who drank monthly but less than weekly, and 2.64 (95% CI 2.34‐2.98) for weekly drinkers.

**Table 2.** Adjusted hazard ratios (HRs) for the association between alcohol consumption and the risk of esophageal cancer in the overall study cohort.

Variables	aHR (95% CI)		*P* value
Alcohol consumption (reference: never drinking)			
Only occasionally	2.02 (1.82‐2.24)		<.001
Every month but less than weekly	1.75 (1.41‐2.18)		<.001
At least once a week	2.64 (2.34‐2.98)		<.001
Sex (reference: male)			
Female	0.22 (0.13‐0.36)		<.001
Age group (y; reference: 30‐49 y)			
50‐54	2.40 (1.89‐3.06)		<.001
55‐59	3.10 (2.42‐3.99)		<.001
60‐64	3.31 (2.52‐4.36)		<.001
65‐69	3.94 (2.93‐5.28)		<.001
70‐74	3.93 (2.78‐5.56)		<.001
75‐79	5.46 (2.49‐11.84)		<.001
Education (reference: uneducated)			
Primary school	1.04 (0.83‐1.31)		.73
Middle school	0.79 (0.60‐1.04)		.09
High school	0.64 (0.44‐0.91)		.01
Technical school or college	0.54 (0.28‐1.01)		.06
University	0.39 (0.16‐0.98)		.04
Marital status (reference: unmarried)			
Married	0.71 (0.38‐1.32)		.27
Divorced or widowed	0.73 (0.39‐1.37)		.33
Household income (¥; reference: ≤¥4999)^a^			
5000‐19,999	0.55 (0.45‐0.67)		<.001
≥20,000	0.35 (0.28‐0.44)		<.001
Area (reference: rural)			
Urban	0.58 (0.48‐0.70)		<.001
Smoking (reference: did not smoke)			
Only occasionally	0.90 (0.61‐1.33)		.58
On most days	1.47 (0.77‐2.79)		.24
Daily	1.59 (1.31‐1.93)		<.001
BMI (reference: normal BMI)			
Underweight	1.25 (1.07‐1.46)		.008
Overweight	0.88 (0.80‐0.97)		.009
Obese	0.89 (0.76‐1.13)		.13
Physical activity (reference: low physical activity)			
Medium	0.85 (0.70‐1.02)		.08
High	0.77 (0.63‐0.93)		.01
Family history of cancer (reference: no family history of cancer)			
Yes	1.54 (1.29‐1.83)		<.001

a An exchange rate of ¥1=US $0.15 is applicable.

### Alcohol Consumption and the Risk of Esophageal Cancer Stratified by Age Group

**Figure 3.**
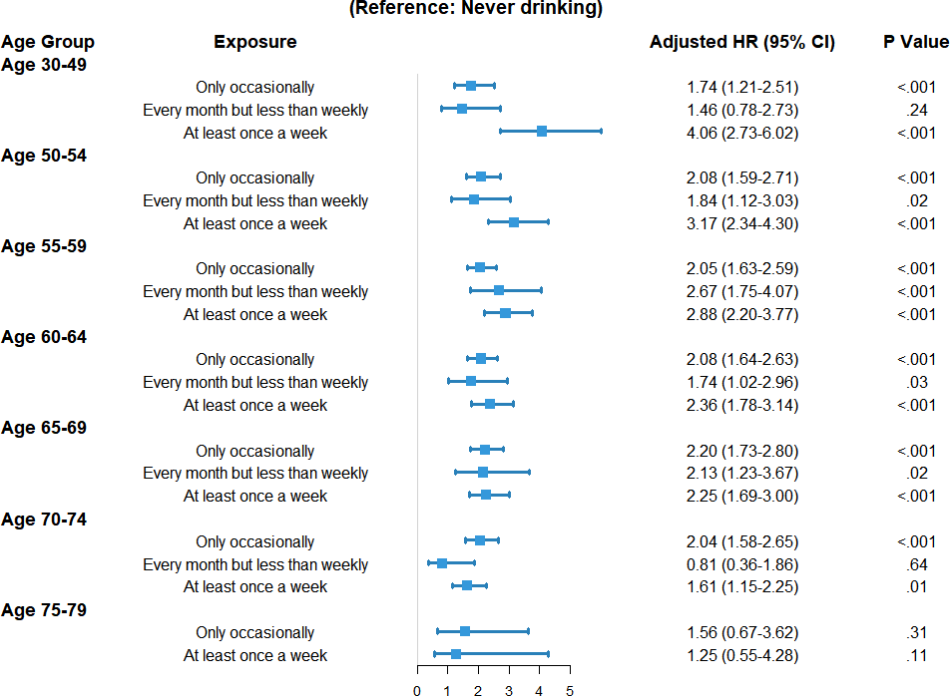
Forest plot of the associations between alcohol consumption and the risk of esophageal cancer, stratified by age group. Estimates for the 75‐79 year age group should be considered preliminary due to limited case numbers. HR: hazard ratio.

Stratified analyses indicated a significant association between weekly drinking and the risk of esophageal cancer among all age groups except the 75‐79 year age group, with a markedly stronger association observed in younger individuals (Figure 3; Figure S1 and Table S1 in Multimedia Appendix 1).

Among participants aged 30‐49 years, weekly drinkers had an aHR of 4.06 (95% CI 2.73‐6.02) compared with never drinkers, and the aHRs for weekly drinkers were 3.17, 2.88, 2.36, 2.25, and 1.61 for the age groups 50‐54, 55‐59, 60‐64, 65‐69, and 70‐74 years, respectively. Significant positive associations for occasional drinking were observed across all age groups from 30‐49 to 70‐74 years, with aHRs ranging from 1.74 to 2.20. For participants who drank every month but less than weekly, significant associations were found primarily in middle-aged and older groups (50‐54, 55‐59, 60‐64, and 65‐69 years), with aHRs ranging from 1.74 to 2.67, although estimates were less stable.

**Figure 4.**
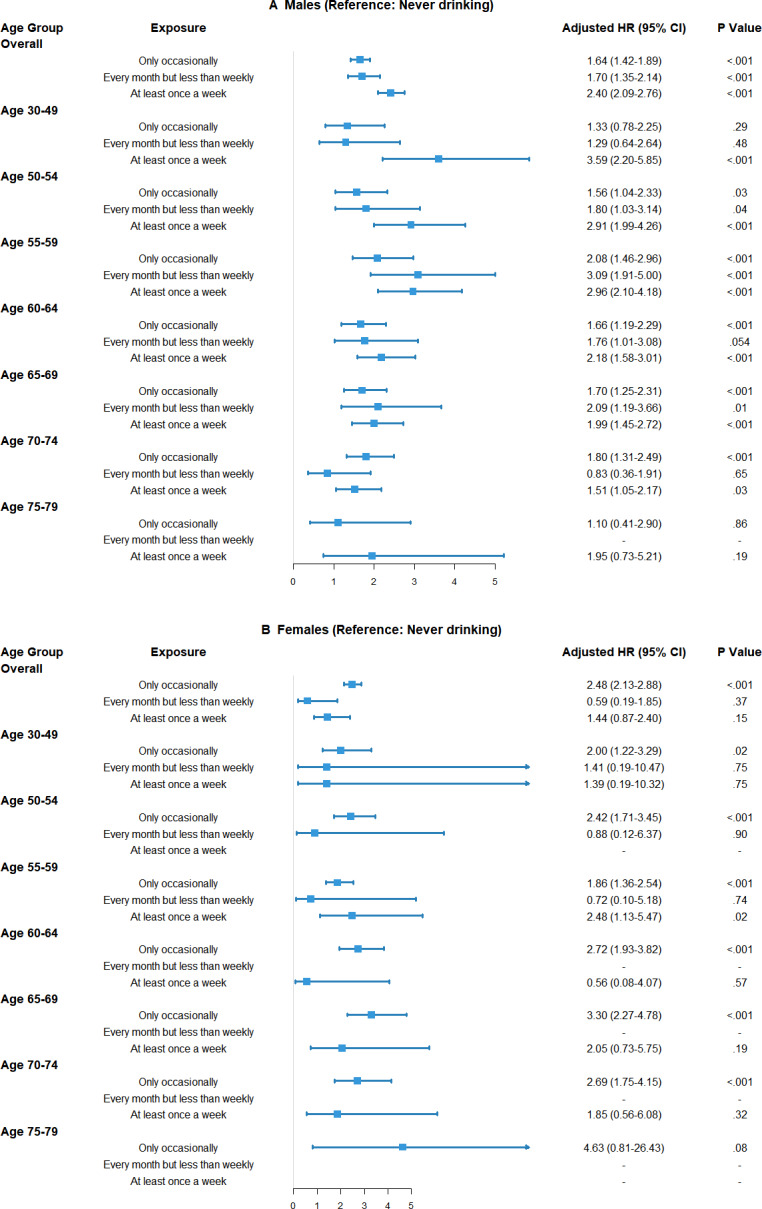
Forest plot of the associations between alcohol consumption and the risk of esophageal cancer, stratified by sex and age group: (A) men and (B) women. Estimates for the 75-79 year age group and for women should be considered preliminary due to limited case numbers. HR: hazard ratio.

In sex-stratified analyses, the association between alcohol consumption and esophageal cancer risk was stronger and more consistent in men than in women (Figure 4). Among men, weekly drinkers had significantly higher risks across all age groups except for those aged 75-79 years compared with never drinkers, with aHRs ranging from 1.51 to 3.59 (all *P*<.05). Occasional drinking also consistently increased the risk in men. In women, the association was less consistent, with wide CIs and mostly nonsignificant results, likely due to low drinking prevalence and fewer female drinkers, which limited statistical power.

Additionally, among participants who reported drinking at least once a week, initiating alcohol consumption at ages 15-24 years was associated with an increased risk of esophageal cancer compared with initiating alcohol consumption after age 45 years (aHR 1.28, 95% CI 1.03-1.64; Figure S2 in Multimedia Appendix 1).

### Sensitivity Analysis

The results from the weighted models were consistent with our primary findings, suggesting that the exclusion criteria did not introduce substantial selection bias or alter our main conclusions. Detailed results are presented in Tables S2-S4 in Multimedia Appendix 1.

## Discussion

Using data from CKB, one of the world’s largest prospective cohorts, we comprehensively evaluated the age-specific associations between alcohol consumption and the risk of developing esophageal cancer. This study had 3 key findings. First, the significant positive association between drinking frequency and the risk of esophageal cancer was consistently observed across age groups. Second, alcohol exposure exhibited a potential age-modifying effect, and the highest relative risk between alcohol consumption and the risk of esophageal cancer was observed in younger adults aged 30‐49 years (aHR 4.06 for weekly vs never drinkers) and gradually declined with increasing age. Finally, among weekly drinkers, a marked increase in incidence from the 30‐49 to the 50‐54 year age group (rate ratio 2.81) highlighted a potential window for targeted prevention.

Our findings reinforce the association between alcohol consumption and the risk of esophageal cancer and further add evidence regarding age- and frequency-specific incidence rates and hazard ratios. The positive associations observed both in the overall cohort and across age groups are consistent with previous studies. For example, data from the Global Burden of Disease (GBD) 2019 study demonstrated that the rates of deaths specifically attributable to alcohol-induced esophageal cancer increased sequentially with age [25]. Notably, the prevalence and high-risk patterns of alcohol use remain a severe public health concern in China across all age groups, serving as an important driver of the associated disease burden. According to 2024 national data for individuals aged 15 years and older, the prevalence of alcohol use is 20.3% in the past 30 days and 27.6% in the past 12 months, with higher rates observed among men and young to middle-aged adults [15]. More than 40% of current drinkers engage in heavy episodic drinking, and a predominant preference was for high-alcohol spirits [15]. These deeply entrenched, high-risk drinking behaviors, combined with our findings of pronounced age-specific vulnerability, underscore a critical need for targeted public health interventions. More efforts are urgently needed to support the implementation of comprehensive primary prevention strategies, including stricter alcohol regulations, increased taxation, and widespread educational campaigns advocating alcohol reduction or cessation [25]. Future studies should aim to refine risk-stratified, age-targeted, and subtype-specific screening strategies to improve the effectiveness of esophageal cancer prevention in China.

Our findings indicate the risk of alcohol consumption on esophageal cancer, particularly among young heavy drinkers. Evidence regarding the increased risk of esophageal cancer associated with alcohol consumption among young people remains limited. The overall prevalence of alcohol consumption peaks among young to middle-aged adults aged 25 to 44 years, reaching a past-year prevalence of 32.5% and a past-month prevalence of 23.2% [15]. A study conducted in a high-risk area in Taixing, China, found that compared with never drinkers, individuals who started drinking between ages 21 and 28 years and those who started before age 21 years had a significantly increased risk of ESCC by 174% and 168%, respectively [16]. Notably, this age-related risk gradient exhibited a clear monotonic increasing trend—the younger the age at which drinking began, the greater the risk increase [16]. A cohort study from Shanghai, China, found that the average age at which esophageal cancer patients started regular drinking (26.0 years) was significantly younger than that of the noncase group (29.8 years) [26]. However, these studies primarily originated from specific high-risk areas for esophageal cancer in China, limiting their generalizability. Analyses based on GBD 2019 data indicated that risk factors such as smokeless tobacco use, low fruit intake, and low vegetable intake contributed more significantly to esophageal cancer risk among younger populations [27]. Therefore, despite the relatively low incidence rate of esophageal cancer among young people, the occurrence of early-onset cancer and other adverse health outcomes necessitates the development of targeted prevention and intervention strategies for adolescents and young adults to effectively reduce alcohol consumption and mitigate the rising future burden of early-onset esophageal cancer.

Our findings reveal a sharp increase in the incidence of esophageal cancer in weekly drinkers between the 30‐49 and 50‐54 year age groups. While global and national epidemiological data indicate that the absolute incidence burden of esophageal cancer generally peaks in much older populations, typically those aged 65 years and older [14,28,29], our results suggest that regular alcohol consumption might accelerate disease onset during midlife. This distinct risk escalation highlights a crucial window of opportunity for targeted clinical interventions. Currently, the National Health Commission of China has launched the 2022 Guideline for Esophageal Cancer Screening, Early Diagnosis, and Early Treatment. This guideline specifies high-risk groups for esophageal cancer, including individuals older than 45 years who live in high-incidence areas, have a family history among first-degree relatives (eg, parents, children, or siblings), exhibit poor lifestyle habits (eg, hot foods, high-salt diets, preserved foods, smoking, and heavy alcohol use), or have preexisting conditions (eg, chronic esophagitis, Barrett esophagus, and a history of precancerous lesions) [30]. The high-incidence areas in China were also attributed to environmental, lifestyle, or socioeconomic factors associated with esophageal cancer risk with geographic clustering [11,13,29]. Similarly, the American College of Gastroenterology and the British Society of Gastroenterology guidelines recommend periodic endoscopy for individuals older than 50 years with additional risk factors [31,32], and these guidelines are primarily focused on EAC. Beyond geographic, familial risk, and preexisting conditions, the findings reemphasize the necessity to aggressively target regular drinkers prior to and during this midlife transition to reduce the risk.

The results of this study should be interpreted in light of several limitations. First, alcohol exposure data were self-reported, and heavy drinking may still be underreported. The CKB implemented multiple strategies to ensure data quality during collection. For example, field interviewers were required to have a medical background and fieldwork experience and to have received standardized training [19]. Second, alcohol consumption data were collected only at baseline, and potential changes during the long-term follow-up may have influenced the risk estimates. However, evidence indicates that lifestyle factors remained relatively stable over an extended period for most CKB participants, suggesting that these potential biases remain within acceptable ranges [18]. Third, the types of esophageal cancer, such as ESCC and EAC, cannot be defined by *ICD-10* codes in the CKB data. Finally, despite our efforts to adjust for various known and suspected confounders, unmeasured or poorly measured factors (eg, dietary risk factors or potential carcinogens) may have an effect.

In conclusion, this study used the large-scale CKB data to provide evidence on the age-modifying associations between alcohol consumption and esophageal cancer risk in China. The findings of a strong relative risk observed in young adults and a marked acceleration during the transition to midlife underscore the importance of developing age-specific public health strategies to mitigate the burden of esophageal cancer, particularly focusing on early alcohol intervention in young populations and enhanced screening in the middle-aged and older adult population.

## Supplementary material

10.2196/92949 Multimedia Appendix 1 Supplementary figures and tables, including incidence rates of esophageal cancer stratified by age group and alcohol consumption, associations between age of alcohol initiation and esophageal cancer risk, and weighted hazard ratio analyses.

## Abbreviations

aHR: adjusted hazard ratio
BEACON: Barrett’s and Esophageal Adenocarcinoma Consortium
CKB: China Kadoorie Biobank
EAC: esophageal adenocarcinoma
ESCC: esophageal squamous cell carcinoma
GBD: Global Burden of Disease
*ICD-10*: I*nternational Classification of Diseases, Tenth Revision*
