# Supplementary material for: Alcohol Consumption and Age-Specific Risk of Esophageal Cancer: Prospective Cohort Study
Source: JMIR Public Health Surveill. 2026 Jun 3;12:e92949. doi: 10.2196/92949 (PMC13233013; doi:10.2196/92949)
Supplement: Multimedia Appendix 1 [file publichealth-v12-e92949-s001.docx]

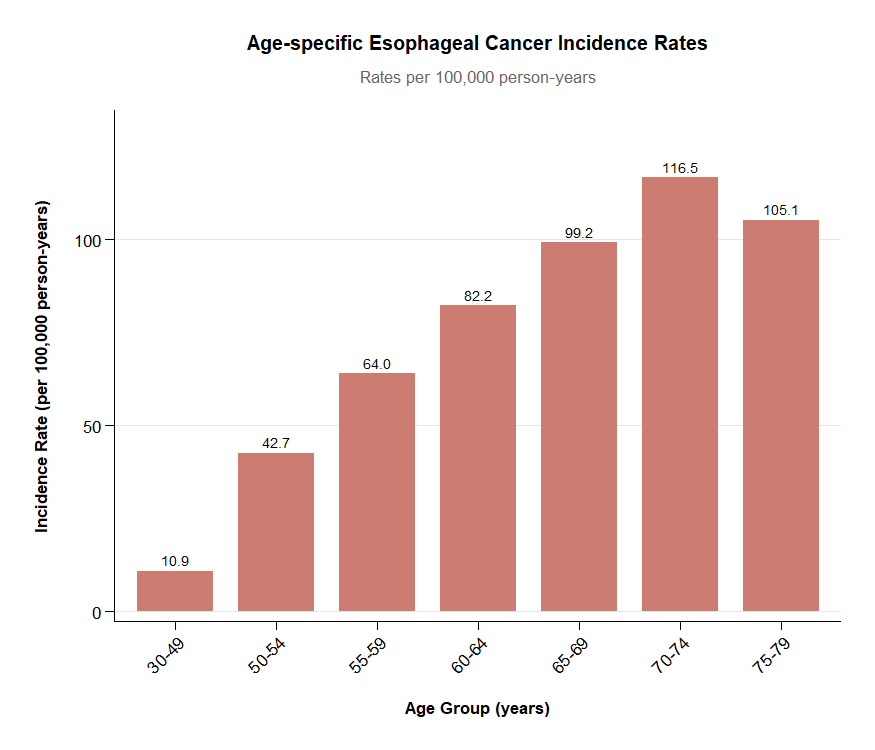


Figure S1. Incidence rates of esophageal cancer stratified by age group (per 100,000 person-years)

Table S1. Incidence rates per 100,000 person-years of esophageal cancer stratified by age group and alcohol consumption

| **Age group_alcohol consumption** | **Person_years** | **Incidence rate** | **95% CI_lower** | **95% CI_upper** |
| --- | --- | --- | --- | --- |
| 30-49_Never | 1,194,206.46 | 3.94 | 2.89 | 5.23 |
| 30-49_Only occasionally | 1,038,529.35 | 9.15 | 7.40 | 11.18 |
| 30-49_Every month but less than weekly | 137,256.80 | 10.20 | 5.57 | 17.11 |
| 30-49_At least once a week | 424,710.29 | 32.73 | 27.51 | 38.64 |
| 50-54_Never | 488,414.40 | 16.79 | 13.35 | 20.84 |
| 50-54_Only occasionally | 348,399.87 | 48.79 | 41.73 | 56.71 |
| 50-54_Every month but less than weekly | 37,453.30 | 58.74 | 36.80 | 88.94 |
| 50-54_At least once a week | 164,106.85 | 92.01 | 77.92 | 107.92 |
| 55-59_Never | 414,389.79 | 27.51 | 22.69 | 33.05 |
| 55-59_Only occasionally | 253,951.41 | 77.57 | 67.12 | 89.20 |
| 55-59_Every month but less than weekly | 24,620.90 | 125.91 | 85.53 | 178.72 |
| 55-59_At least once a week | 118,106.48 | 129.54 | 109.83 | 151.78 |
| 60-64_Never | 301,128.46 | 41.51 | 34.55 | 49.46 |
| 60-64_Only occasionally | 165,377.39 | 115.49 | 99.69 | 133.09 |
| 60-64_Every month but less than weekly | 14,584.41 | 116.56 | 67.86 | 186.64 |
| 60-64_At least once a week | 77,972.79 | 143.64 | 118.27 | 172.84 |
| 65-69_Never | 248,266.90 | 48.34 | 40.07 | 57.80 |
| 65-69_Only occasionally | 123,952.61 | 141.99 | 121.79 | 164.59 |
| 65-69_Every month but less than weekly | 10,163.73 | 167.26 | 97.38 | 267.82 |
| 65-69_At least once a week | 59,301.20 | 172.00 | 140.24 | 208.80 |
| 70-74_Never | 164,485.44 | 63.84 | 52.21 | 77.28 |
| 70-74_Only occasionally | 78,213.30 | 179.00 | 150.57 | 211.23 |
| 70-74_Every month but less than weekly | 6,747.88 | 103.74 | 41.56 | 213.75 |
| 70-74_At least once a week | 36,841.67 | 176.43 | 136.16 | 224.88 |
| 75-79_Never | 17,995.73 | 66.68 | 34.42 | 116.49 |
| 75-79_Only occasionally | 8,573.59 | 139.96 | 72.24 | 244.51 |
| 75-79_Every month but less than weekly | 695.50 | 0.00 | 0.00 | 530.40 |
| 75-79_At least once a week | 3,542.97 | 225.80 | 97.23 | 444.94 |


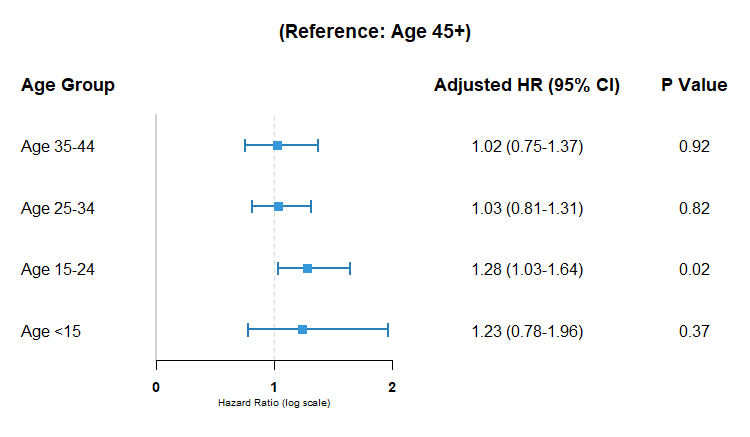


Figure S2. Forest plot of the associations between the age of alcohol initiation and the risk of esophageal cancer among participants who usually drank at least once a week

**Table S2. Weighted hazard ratios for the association between alcohol consumption and the risk of esophageal cancer in the overall study cohort**

| Variables | HR | 95%_CI | P_value |
| --- | --- | --- | --- |
| Alcohol consumption (vs. Never) |  |  |  |
| Only occasionally | 2.05 | 1.84 - 2.28 | <0.001 |
| Every month but less than weekly | 1.79 | 1.44 - 2.22 | <0.001 |
| At least once a week | 2.68 | 2.38 - 3.01 | <0.001 |
| Sex (vs. Male) |  |  |  |
| Female | 0.41 | 0.37 - 0.46 | <0.001 |
| Age_group (vs. Age_30-49) |  |  |  |
| Age_50-54 | 2.38 | 1.90 - 2.93 | <0.001 |
| Age_55-59 | 3.64 | 2.98 - 4.40 | <0.001 |
| Age_60-64 | 4.51 | 3.69 - 5.47 | <0.001 |
| Age_65-69 | 5.51 | 4.49 - 6.71 | <0.001 |
| Age_70-74 | 6.53 | 5.26 - 8.07 | <0.001 |
| Age_75-79 | 6.03 | 3.78 - 9.33 | <0.001 |
| BMI_group (vs. Normal) |  |  |  |
| Underweight | 1.25 | 1.07 - 1.46 | 0.008 |
| Overweight | 0.88 | 0.80 - 0.97 | 0.009 |
| Obese | 0.89 | 0.76 - 1.13 | 0.132 |
| Marital status (vs. Unmarried) |  |  |  |
| Married | 0.71 | 0.38 - 1.32 | 0.274 |
| Divorce / widow | 0.73 | 0.38 - 1.38 | 0.326 |
| Smoking (vs. Did not smoke) |  |  |  |
| Only occasionally | 0.91 | 0.76 - 1.09 | 0.292 |
| On most day | 1.14 | 0.80 - 1.53 | 0.486 |
| Daily | 1.45 | 1.23 - 1.83 | <0.001 |
| Area (vs. Rural) |  |  |  |
| Urban | 0.33 | 0.30 - 0.37 | <0.001 |
| Education (vs. Uneducated) |  |  |  |
| Primary school | 0.93 | 0.84 - 1.04 | 0.210 |
| Middle school | 0.71 | 0.61 - 0.81 | <0.001 |
| High school | 0.58 | 0.48 - 0.71 | <0.001 |
| Technical school / college | 0.46 | 0.30 - 0.71 | <0.001 |
| University | 0.22 | 0.11 - 0.45 | <0.001 |
| Income (vs. Low) |  |  |  |
| Medium | 0.78 | 0.71 - 0.87 | <0.001 |
| High | 0.43 | 0.38 - 0.49 | <0.001 |
| MET (vs. Low) |  |  |  |
| Medium | 0.71 | 0.64 - 0.78 | <0.001 |
| High | 0.58 | 0.52 - 0.65 | <0.001 |
| Family history of cancer (vs. No) |  |  |  |
| Yes | 2.20 | 2.02 - 2.40 | <0.001 |

**Table S3. Weighted hazard ratios for associations between alcohol consumption and the risk of esophageal cancer, stratified by age group.**

| Variables | HR | 95%_CI | P_value |
| --- | --- | --- | --- |
| *Age 30-49* |  |  |  |
| Alcohol consumption (vs. Never) |  |  |  |
| Only occasionally | 1.78 | 1.25 - 2.55 | <0.001 |
| Every month but less than weekly | 1.49 | 0.81 - 2.76 | 0.216 |
| At least once a week | 4.12 | 2.82 - 6.09 | <0.001 |
| *Age 50-54* |  |  |  |
| Alcohol consumption (vs. Never) |  |  |  |
| Only occasionally | 2.12 | 1.63 - 2.76 | <0.001 |
| Every month but less than weekly | 1.88 | 1.16 - 3.09 | 0.014 |
| At least once a week | 3.22 | 2.39 - 4.36 | <0.001 |
| *Age 55-59* |  |  |  |
| Alcohol consumption (vs. Never) |  |  |  |
| Only occasionally | 2.09 | 1.67 - 2.63 | <0.001 |
| Every month but less than weekly | 2.71 | 1.79 - 4.12 | <0.001 |
| At least once a week | 2.93 | 2.24 - 3.82 | <0.001 |
| *Age 60-64* |  |  |  |
| Alcohol consumption (vs. Never) |  |  |  |
| Only occasionally | 2.07 | 1.65 - 2.61 | <0.001 |
| Every month but less than weekly | 1.81 | 1.42 - 2.31 | <0.001 |
| At least once a week | 2.72 | 2.32 - 3.12 | <0.001 |
| *Age 65-69* |  |  |  |
| Alcohol consumption (vs. Never) |  |  |  |
| Only occasionally | 2.03 | 1.53 - 2.63 | <0.001 |
| Every month but less than weekly | 1.72 | 1.32 - 3.65 | <0.001 |
| At least once a week | 2.63 | 1.73 - 3.03 | <0.001 |
| *Age 70-74* |  |  |  |
| Alcohol consumption (vs. Never) |  |  |  |
| Only occasionally | 1.98 | 1.43 - 2.63 | <0.001 |
| Every month but less than weekly | 0.85 | 0.39 - 1.92 | 0.508 |
| At least once a week | 2.53 | 2.13 - 3.03 | <0.001 |
| *Age 75-79* |  |  |  |
| Alcohol consumption (vs. Never) |  |  |  |
| Only occasionally | 1.65 | 0.78 - 3.75 | 0.284 |
| At least once a week | 1.29 | 0.59 - 4.33 | 0.101 |

**Table S4. Weighted hazard ratios for associations between the age of alcohol initiation and the risk of esophageal cancer among participants who usually drank at least once a week**

| Variables | HR | 95%_CI | P_value |
| --- | --- | --- | --- |
| **Age of alcohol initiation** (vs. Never) |  |  |  |
| Age 35-44 | 1.02 | 0.76 - 1.38 |  |
| Age 25-34 | 1.04 | 0.82 - 1.32 | <0.001 |
| Age 15-24 | 1.29 | 1.04 - 1.66 | 0.115 |
| Age <15 | 1.25 | 0.79 - 1.98 | 0.349 |
